# The use of negative pressure wound therapy following stoma reversal: a systematic review and meta-analysis of randomized controlled trials

**DOI:** 10.1007/s00384-025-04865-2

**Published:** 2025-03-20

**Authors:** Caroline Drumm, Ben Creavin, Iva Pranjic Previsic, Maeve O’Neill, John Larkin, Brian J. Mehigan, Dara Kavanagh, Paul McCormick, Michael Eamon Kelly

**Affiliations:** 1 Department of Surgery, St. James’s Hospital, Dublin, Ireland; 2 Department of Surgery, Tallaght University Hospital, Dublin, Ireland

**Keywords:** Negative pressure, NPWT, Stoma reversal, Colorectal surgery

## Abstract

**Introduction:**

Stoma reversal is a contaminated surgery with many patients experiencing significant wound complications that contribute to patient morbidity. It is believed that the use of prophylactic negative pressure wound therapy (NPWT) may enhance wound healing and help reduce the risk of developing surgical site infections (SSI). However, there is conflicting research regarding its effectiveness following stoma reversal. Our systematic review aims to evaluate the available randomized data to determine if the use of prophylactic NPWT after stoma reversal improves the duration of wound healing and reduces rates of postoperative complications.

**Methods:**

A comprehensive search of literature published up to January 2025 was conducted using the following databases: PubMed, Embase, Medline, and Cochrane Library. The included trials were randomized controlled trials that investigated the effect of NPWT following stoma reversal. The primary outcome was the time to complete wound healing. Secondary outcomes included the incidence of wound complications, SSI, hematomas, and the length of hospital stay.

**Results:**

Six randomised control trials were included, with 332 patients, of which 171 of these underwent NPWT. There was a significant reduction in time to complete wound healing (OR − 2.53, 95% CI − 3.82 to − 1.24, *p* = 0.0001, *I*^2^ = 45%) and wound healing at 42 days (OR 0.36, 95% CI 0.14 − 0.88, *p* = 0.03, *I*^2^ = 0%) in the NPWT group. There was no significant difference in any wound complications (OR 0.72, 95% CI 0.23–2.28, *p* = 0.58, *I*^2^ = 42%), SSI rates (OR 0.95, 95% CI 0.27–3.29, *p* = 0.94, *I*^2^ = 38%) or haematoma rates (OR 0.21, 95% CI 0.03–1.27, *p* = 0.09, *I*^2^ = 0%) between the groups. There was no significant difference in length of stay (OR − 0.02, 95% CI − 1.21–1.18, *p* = 0.98, *I*^2^ = 66%).

**Conclusion:**

The use of NPWT after stoma reversal significantly reduces the time needed for complete wound healing while maintaining a comparable rate of wound complications and length of hospital stay. Therefore, NPWT may be valuable in optimizing postoperative recovery and enhancing patient outcomes.

## Introduction

Stoma reversal is a contaminated surgery that often leads to significant wound complications, such as prolonged wound healing and surgical site infections (SSI) [1]. These factors greatly contribute to patient morbidity, resulting in increased post-operative pain, extended hospital stays, readmissions, and the necessity for repeat procedures [2]. For these reasons, many surgeons leave the skin open with daily dressings for several months to obviate the risk of SSI. It is now believed that the use of prophylactic negative pressure wound therapy (NPWT) may enhance wound healing and reduce the risk of developing SSI [3–5].

NPWT is a system that promotes “angiogenesis, extracellular matrix remodelling, and granulation tissue deposition,” thereby encouraging wound healing [6]. It is often utilized in complicated surgical incisions, including infections, seromas, and non-healing ulcers. However, there is limited evidence regarding the prophylactic use of such systems to enhance wound healing and decrease the risk of complications. Ultimately, the advantages of using NPWT prophylactically after the reversal of an ileostomy or colostomy remain unclear, with conflicting research surrounding this topic [4, 5, 7–10].

There have been two previous meta-analyses published to date on this topic; however, both included randomized and non-randomized studies. Kisielewski et al. demonstrated that the use of incisional NPWT can reduce the risk of SSIs as well as other complications, such as hematomas, seromas, dehiscence, and fistula formation, with no increase in the length of hospital stay [11]. Zhu et al. reviewed nine studies, including randomized controlled trials and retrospective or prospective observational studies, and concluded that NPWT reduces the risk of SSI without a significant reduction in other wound complications [3]. Further assessment was necessary following the aforementioned studies due to the heterogeneity of the included studies and the partly conflicting results.

In light of these mixed results, our study aims to assess only randomized data to determine whether the use of prophylactic NPWT following stoma reversal improves the duration of wound healing and reduces rates of post-operative complications. This data will be instrumental in developing optimized post-operative protocols, potentially enhancing patient outcomes and reducing the associated healthcare costs of SSIs and prolonged recovery times.

## Methods

A comprehensive search of literature published up to January 2025 was performed using the following databases: PubMed, Embase, Medline, and the Cochrane Library. The search terms included stoma reversal, stoma closure, and negative pressure therapy. This search was conducted in accordance with the Preferred Reporting Items for Systematic Reviews and Meta-Analyses (PRISMA) 2020 guidelines [12].

### Inclusion criteria

The included trials were human-based randomized controlled trials that investigated the effect of NPWT following the reversal of ileostomy or colostomy for benign or oncological indications. The RCTs must have included one of the following outcomes: time to complete wound healing, incidence of wound complications, incidence of SSI, and/or hematomas.

Two collaborators examined potentially eligible studies, initially conducting a title and abstract review followed by a full-text review to determine eligibility for inclusion. Reference lists were cross-referenced to ensure that no studies were overlooked. Any discrepancies were resolved through joint agreement between both collaborators. All non-randomized trials were excluded.

### Extraction of data

Characteristics of the baseline study were extracted, including the number of patients involved, patient demographics, details of interventions and controls, the follow-up period, and outcomes. The primary outcome was the time taken to achieve complete wound healing. Secondary outcomes included the incidence of wound complications, SSI, hematomas, and the duration of the hospital stay.

### Statistical analysis

Statistical analysis was performed using Review Manager version 5.3 (The Nordic Cochrane Centre, The Cochrane Collaboration, Copenhagen, Denmark). Binary outcome data was reported as odds ratios (OR) with 95% confidence intervals (CI) estimated using the Mantel–Haenszel method. An OR of below 1 favors NPWT. For continuous data, standard mean difference (MDs) and 95% CI were estimated using inverse-variance weighting. Outcome measures were recorded as mean (s.d) or median (i.q.r) based on available data. Heterogeneity was assessed by means of *I*^2^ statistics, with considerable heterogeneity considered with a value of higher than 0%. Pooled estimates of differences were calculated using random-effects models, accounting for potential interstudy heterogeneity. Sensitivity analyses were carried out where appropriate. *P* < 0.05 was considered significant. Quality of included RCTs was assessed independently by two authors using the JADAD/Oxford quality scoring system.

## Results

**Fig. 1:**
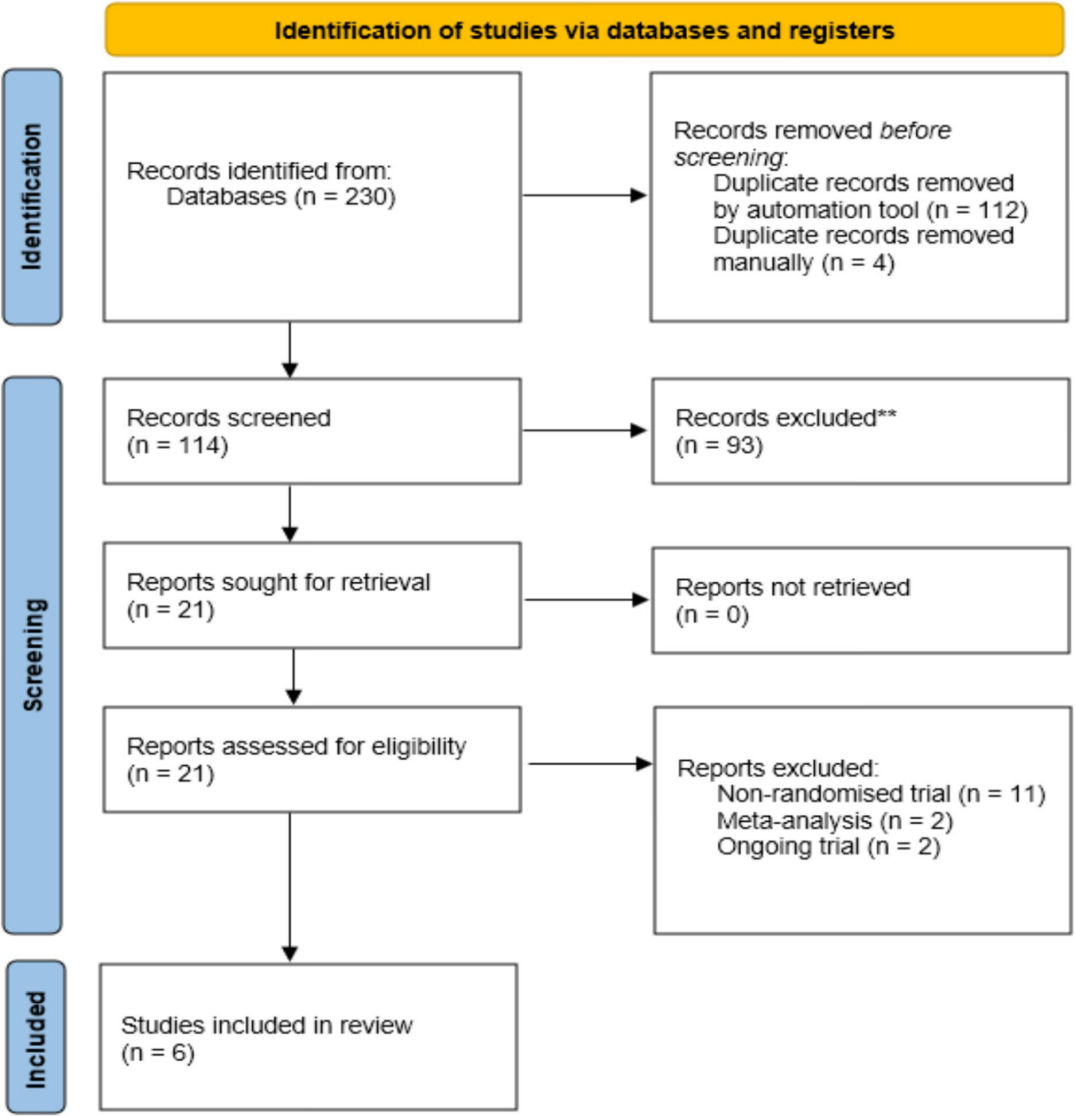
PRISMA flow diagram (12)

A total of 230 articles were initially identified in the database search. Upon full-text screening, six publications met the predefined inclusion and exclusion criteria (Fig. 1). All studies were randomized trials published between 2016 and 2024. Each study was comparative, reporting on NPWT versus no NPWT in stoma reversals. The details of skin closure methods, type and duration of NPWT used, and details of controls are outlined in Table 1. All reviewers agreed 100% when reviewing the extracted data. The characteristics and quality of the studies assessed by the JADAD score are outlined in Table 2.

**Table 1:** Characteristics of NPWT

	**Uchino et al. (2016)**	**Wierdak et al. (2020)**	**Kojima et al. (2021)**	**Carrano et al. (2021)**	**Kang et al. (2023)**	**Tiang et al. (2024)**
Skin closure method	PSC^1^	Complete skin closure	PSC	PSC	PSC	PSC
NPWT device	PICO	NANOVA	PICO	PICO	PICO	SNap
Duration of NPWT (days)	14	7	7–14	7	Dependent on wound healing progress	7
Control	Simple dressing	Simple dressing	Simple dressing	Cavity packed, simple dressing	Simple dressing	Simple dressing

^1^*PSC* purse string closure

**Table 2:** JADAD scores

	**Uchino et al. (2016)**	**Wierdak et al. (2020)**	**Kojima et al. (2021)**	**Carrano et al. (2021)**	**Kang et al. (2023)**	**Tiang et al. (2024)**
JADAD score	3	3	3	3	3	3

### Demographics

The analysis included 332 patients, of whom 171 underwent NPWT. A total of 205 males were involved across both groups. The indications for initial surgery comprised both benign and malignant conditions. The mean age in the NPWT group was 61.02 ± 14.4 years, while it was 59.31 ± 13.7 years for the other group. There was no difference in BMI between the groups. Patient demographics are presented in Table 3.

**Table 3:** Patient demographics

	**Uchino et al. (2016)**		**Wierdak et al. (2020)**		**Kojima et al. (2021)**		**Carrano et al. (2021)**		**Kang et al. (2023)**		**Tiang et al. (2024)**	
	*NPWT*	Control	NPWT	Control	NPWT	Control	NPWT	Control	NPWT	Control	NPWT	Control
Sample size	28	31	35	36	20	10	50	48	18	16	20	20
Male	17	23	24	20	12	6	35	31	9	8	10	10
Age at surgery^1^	48.1	40.4	61.6	62.4	69	64	56.3	55.1	66.5	72	55.7	62
Oncological indication for initial surgery	0	0	35	36	-	-	28	26	-	-	11	14
Ileostomy reversal	-	-	-	-	20	10	41	38	12	10	-	-
BMI^2^	19.8	19.7	26.2	26.2	21.1	22.2	23.8	23.5	25.4	23.7	27.6	26.1
ASA grade 1/2	26	30	23	25	-	-	47	44	18	16	20	20
ASA grade 3 +	2	1	12	11	-	-	3	4	0	0	0	0
Smoking	0	0	5	6	-	-	9	5	1	1	-	-

^1^Age in years

^2^ kg/m^2^

### Time to wound healing

**Fig. 2:**
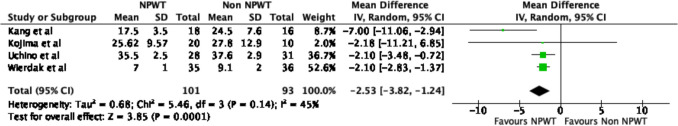
Time to wound healing

**Fig. 3:**
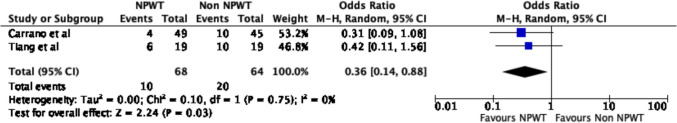
Complete wound healing within 42 days

All six studies reported on the time to wound healing rates. Four studies detailed the time to wound healing in days, with a significant difference noted in the NPWT group (OR − 2.53, 95% CI − 3.82 to − 1.24, *p* = 0.0001, *I*^2^ = 45%) (Fig. 2). Two studies examined completeness of wound healing within 42 days post-surgery. Once again, a significant difference was observed in the NPWT group (OR 0.36, 95% CI 0.14–0.88, *p* = 0.03, *I*^2^ = 0%) (Fig. 3).

### Wound outcomes

**Fig. 4:**
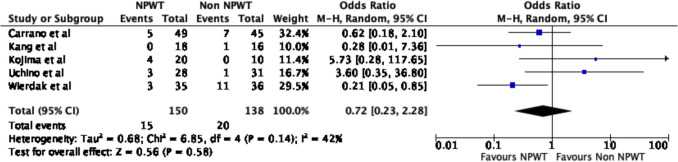
Wound complications

**Fig. 5:**
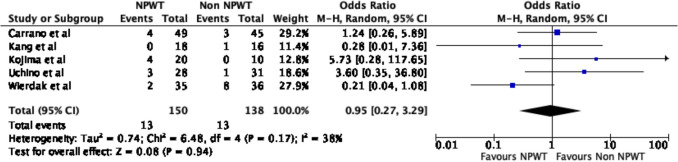
Surgical site infections

**Fig. 6:**
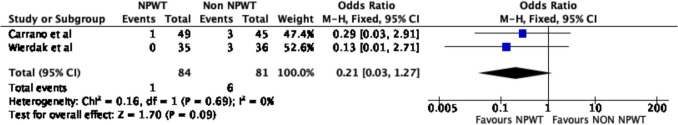
Haematomas

Five of the six studies reported on wound outcomes. There were no significant differences in any wound complications between the NPWT and non-NPWT groups (OR 0.72, 95% CI 0.23–2.28, *p* = 0.58, *I*^2^ = 42%) (Fig. 4). Additionally, there were no differences in SSI rates (OR 0.95, 95% CI 0.27–3.29, *p* = 0.94, *I*^2^ = 38%) (Fig. 5) or hematoma rates (OR 0.21, 95% CI 0.03–1.27, *p* = 0.09, *I*^2^ = 0%) (Fig. 6) between the groups.

### Length of Stay

**Fig. 7:**
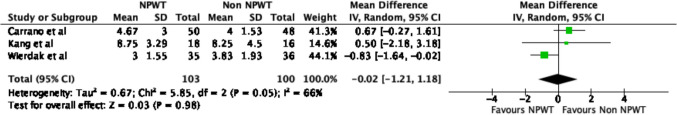
Length of stay

There was no significant difference in length of stay (OR − 0.02, 95% CI − 1.21 to 1.18, *p* = 0.98, *I*^2^ = 66%) (Fig. 7).

### Sub-group analysis of PSC

**Fig. 8:**
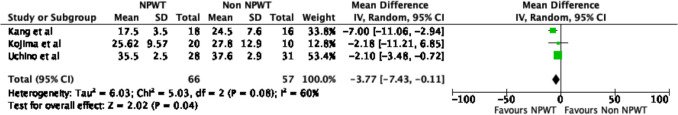
Time to wound healing following PSC

**Fig. 9:**
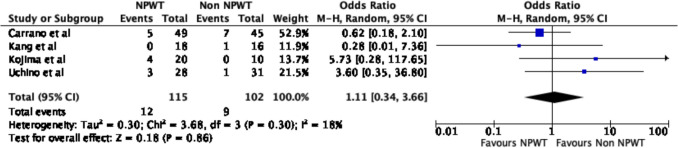
Wound complications following PSC

**Fig. 10:**
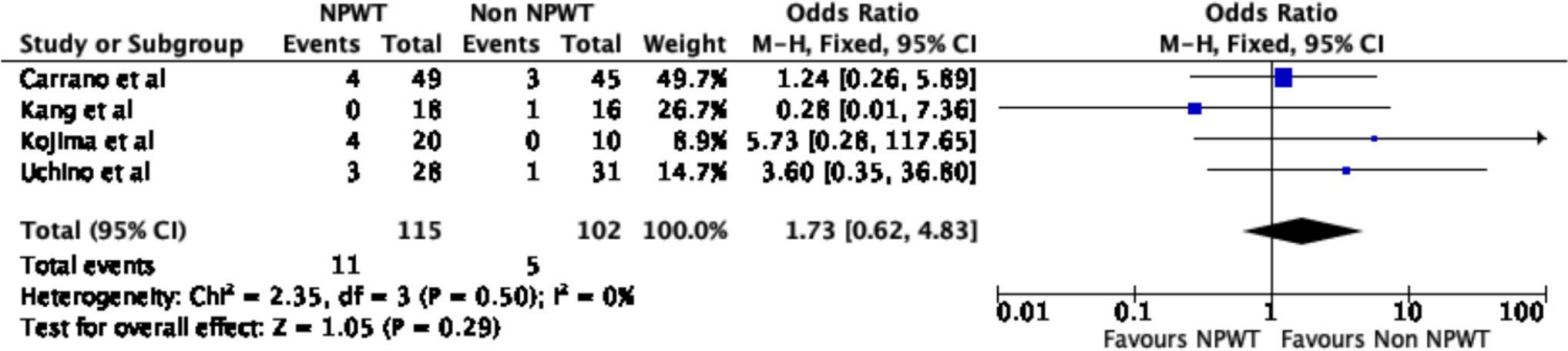
Surgical site infection following PSC

A sub-group analysis was conducted, including only the studies that utilised PSC as the sole method of skin closure. Similarly, there was a significant reduction in time to wound healing between the NPWT group and non-NPWT group (OR − 3.77, 95% CI − 7.43–0.11, *p* = 0.04, *I*^2^ = 60%) (Fig. 8). There was no significant difference in overall wound complications (OR 1.11, 95% CI 0.34–3.66, *p* = 0.86, *I*^2^ = 18%) (Fig. 9) or surgical site infections alone (OR 1.73, 95% CI 0.62–4.83, *p* = 0.29, *I*^2^ = 0%) (Fig. 10).

## Discussion

This systematic review of randomized trials demonstrated that using NPWT after stoma reversal significantly shortened the duration of wound healing, without significant differences in rates of wound complications, such as SSI and hematomas, or length of hospital stay. A subgroup analysis that included only studies using PSC yielded similar results.

Stoma reversal is conducted to restore bowel continuity, enhance patients’ quality of life, and minimize the psychosocial impacts associated with living with a stoma. However, these procedures can have complications, including delayed wound healing, SSI, and the development of hematomas and seromas. Fortunately, research indicates a generally low incidence of postoperative complications following stoma reversal [1, 13]. Nevertheless, these complications can result in longer hospital stays, increased healthcare expenses, potential delays in adjuvant cancer treatments, and psychological effects on patients stemming from these issues [14]. Thus, minimizing any postoperative complications is crucial.

Historically, stoma sites were closed in a linear manner using interrupted sutures. During that period, the incidence of wound complications was significantly higher, with rates of SSI reaching up to 41% [15]. In 1997, Banerjee introduced a new technique known as purse-string closure (PSC), which was associated with reduced scarring and SSI rates [16]. Subsequent studies demonstrated that this technique lowers the incidence of post-operative wound infections [17]. However, this technique also introduced a new challenge: prolonged wound healing [18]. NPWT could provide a solution to this issue following PSC, as this review revealed a statistically significant decrease in the time to achieve complete wound healing. Only one study evaluated the optimal duration of NPWT, finding that 3 to 10 days was ideal for maximizing the wound reduction rate after ileostomy closure [10]. The clinical benefits were evident in a reduced need for dressing clinic visits and outpatient consultations, as well as enhanced post-operative recovery and quality of life for these patients.

The effectiveness of NPWT in managing wound complications is well recognized. However, its use for prevention is less common. This study highlights the safety of NPWT following stoma reversal, showing no increase in post-operative wound complications, such as SSI and haematomas. In fact, although not statistically significant, the rates of wound complications in the NPWT group were lower, therefore favoring its use. To achieve statistical significance in demonstrating a reduction in wound complications with the use of NPWT following stoma reversal, a sample size of 222 participants wound be required. This is based off the PRIC protocol, a RCT currently underway in our center, which hypothesizes a 60% reduction in SSI rates following the routine use of NPWT. Under these assumptions, this sample size will achieve a power of 1-*β* = 0.80 and level *α* = 0.05, incorporating a margin of error and a 10% drop out rate [19].

Regarding SSI, this study did not elicit a statistically significant result. Five of the six RCTs utilized PSC technique for stoma site closure. As discussed, this technique has been shown to significantly reduce SSIs following stoma reversals [17]. The number of SSIs observed in the included studies was low, potentially indicating that these studies were not adequately powered to produce statistically significant results. Consequently, prophylactic efficacy could not be established. This is underscored by examining Wierdak et al., which showed a statistically significant reduction in the use of NPWT following the linear closure of stoma sites with interrupted sutures made from non-absorbable material [4].

It is possible that there is a subgroup of patients for whom the use of NPWT is more effective. This has previously been demonstrated in other scenarios, with multiple studies evaluating the use of prophylactic NPWT in high-risk patients undergoing emergency and elective open abdominal surgery, resulting in a reduced incidence of SSI in these high-risk patients [20, 21]. In other specialties, such as Oncoplastic and Breast Reconstruction Surgery, prophylactic NPWT has been shown to decrease wound complications and associated healthcare costs following reconstruction surgeries. This included high-risk patients, such as those who are overweight or obese, current smokers, those with T1 or T2 diabetes, individuals who have had previous radiotherapy, neoadjuvant chemotherapy, or those currently using corticosteroids [22]. In our review, Wierdek et al. demonstrated this in cancer patients, the majority of whom were post-neoadjuvant chemotherapy and/or radiotherapy patients—groups that are at an increased risk of postoperative complications. They experienced a significantly reduced incidence of SSI and other wound complications [4]. Other studies specifically excluded high-risk patients, which may have further reduced the incidence of complications and, consequently, the power of this study to yield statistically significant results [10].

Cosmetic outcomes were also evaluated in several RCTs. Carrano et al. employed a visual analogue scale (VAS) to measure aesthetic results 7 days post-operation. They observed significantly better outcomes in the NPWT group compared to the control group [8]. Two other RCTs reported no differences in cosmetic-related outcomes—one also utilizing a VAS at days 14 and 42, while the other applied POSAS (Patient and Observer Scar Assessment Scale) scores at 1-month post-operation [5, 9].

Post-operative pain is a common complication of any surgical procedure, one that can extend the length of hospital stay, require readmission for management and investigation, and can dramatically affect recovery with poor respiratory and mobility outcomes. One randomized controlled trial aimed to assess whether the use of NPWT improved post-operative pain scores. It produced statistically significant results, with reduced patient-reported pain on post-operative days 3 and 7 [8]. Therefore, the use of NPWT could enhance patient experience and recovery.

Only one RCT addressed cost-effectiveness in its study [5]. It showed no difference in costs between the NPWT and control groups. However, this only considered the cost of dressings themselves. While this is reassuring, there are clearly several other factors to consider for a full and comprehensive assessment of cost differences. These include length of stay, treatment of post-operative wound infections, dressing clinic visits, absenteeism at work, and outpatient consultations, all of which would be necessary for both groups. An initial high-cost burden is expected with the use of NPWT; yet, overall costs may decrease with a reduced time to complete wound healing, leading to fewer dressing changes and outpatient reviews. Additionally, this study found no significant difference in length of stay.

The main strength of this study lies in the inclusion of only RCTs, which represents the highest level of evidence. However, there are several limitations. First, the sample size was small, consisting of 332 patients across six RCTs that were included and analysed. Second, heterogeneity was observed among the included studies, as different skin closure methods and varying outcomes with distinct measurement tools were used. A sub-group analysis was conducted including only the studies who utilized PSC in an attempt to reduce heterogeneity. This analysis yielded similar results to the main group analysis. It was not possible to combine and analyze outcomes related to cosmetic results, pain, and cost-effectiveness, since different assessment tools were employed in various studies. Although these outcomes were included as secondary measures, they were not necessarily powered to yield statistically significant results. Regarding the quality of the included studies, all scored 3 out of 5 on the JADAD scale, largely due to the inability to implement blinding given the nature of the intervention being investigated. Finally, it remains unknown what the optimal NPWT device is or which device settings might yield the best results. There is also no clarity on the optimal duration of use, as current practices depend on surgeon preference without established guidelines or protocols. Additionally, it is still unclear whether a specific cohort of higher risk patients may gain greater benefits from its use, leading to improved outcomes for that group as well as more cost-effective use of NPWT.

## Conclusion

The use of NPWT after stoma reversal significantly reduces the time to complete wound healing. This suggests that NPWT may be a useful adjunct in postoperative recovery. Despite these encouraging findings, several important questions remain unanswered, including the optimal device, device settings and duration of therapy, cost-effectiveness, and ideal patient selection criteria.

## Data Availability

No datasets were generated or analysed during the current study.
